# In Vitro Evaluation of Farnesyltransferase Inhibitor and its Effect in Combination with 3-Hydroxy-3-Methyl-Glutaryl-CoA Reductase Inhibitor against *Naegleria fowleri*

**DOI:** 10.3390/pathogens9090689

**Published:** 2020-08-22

**Authors:** Hye Jee Hahn, Anjan Debnath

**Affiliations:** Center for Discovery and Innovation in Parasitic Diseases, Skaggs School of Pharmacy and Pharmaceutical Sciences, University of California San Diego, La Jolla, CA 92093, USA; lilyh814@gmail.com

**Keywords:** *Naegleria fowleri*, free-living amoeba, primary amoebic meningoencephalitis, lonafarnib, pitavastatin, farnesyltransferase, 3-hydroxy-3-methyl-glutaryl-CoA reductase

## Abstract

Free-living amoeba *Naegleria fowleri* causes a rapidly fatal infection primary amebic meningoencephalitis (PAM) in children. The drug of choice in treating PAM is amphotericin B, but very few patients treated with amphotericin B have survived PAM. Therefore, development of efficient drugs is a critical unmet need. We identified that the FDA-approved pitavastatin, an inhibitor of HMG Co-A reductase involved in the mevalonate pathway, was equipotent to amphotericin B against *N. fowleri* trophozoites. The genome of *N. fowleri* contains a gene encoding protein farnesyltransferase (FT), the last common enzyme for products derived from the mevalonate pathway. Here, we show that a clinically advanced FT inhibitor lonafarnib is active against different strains of *N. fowleri* with EC_50_ ranging from 1.5 to 9.2 µM. A combination of lonafarnib and pitavastatin at different ratios led to 95% growth inhibition of trophozoites and the combination achieved a dose reduction of about 2- to 28-fold for lonafarnib and 5- to 30-fold for pitavastatin. No trophozoite with normal morphology was found when trophozoites were treated for 48 h with a combination of 1.7 µM each of lonafarnib and pitavastatin. Combination of lonafarnib and pitavastatin may contribute to the development of a new drug regimen for the treatment of PAM.

## 1. Introduction

*Naegleria* spp. are protozoa belonging to the family Vahlkampfiidae and class Heterolobosea. Like the rest of their class, they are amphizoic and they do not need to infect a host to survive. They can live by feeding themselves mostly on bacteria [1] and can transform into a flagellate form if the ionic concentration in the milieu changes. The amoeba can also turn into a cyst stage to survive adverse conditions such as low food supply [2]. *Naegleria* species have been known for long time, but it was not until around 50 years ago that the human pathogenic *Naegleria fowleri* was discovered [3]. The pathology of *N. fowleri* is caused by the trophozoites through the release of cytolytic molecules like acid hydrolases, phospholipases, neuraminidases and phospholipolytic enzymes [4], leading to the brain tissue destruction for which *N. fowleri* is commonly known as brain-eating amoeba [2].

*N. fowleri* causes a fulminating meningoencephalitis known as primary amoebic meningoencephalitis (PAM). Most of the infections have been reported in healthy children and young adults following recreational water activities, especially in warm water. *N. fowleri* is thermophilic and can tolerate temperatures up to 45°C [2]. The fact that the infection is reported more from subtropical and temperate countries than tropical areas may be due to the availability of better diagnostics in these countries. According to the Centers for Disease Control and Prevention (CDC), 145 cases were documented from 1962 to 2019 in the Unites States and only four people survived. Considering a mortality rate with >97%, the difficulty in diagnosis—with 75% of the cases are diagnosed after the death of the patient—and that children are the most affected, PAM causes a serious problem for public health both in developed and developing countries [5,6]. Furthermore, it might be possible in a not-so-distant future that global warming may increase the chances of people encountering *N. fowleri* and contracting PAM [7]. 

Symptoms of PAM start 1–9 days after exposure to *N. fowleri*-contaminated water. Symptoms during stage 1 of PAM include a severe frontal headache, nausea and vomiting, and a fever. Stage 2 symptoms display a stiff neck; altered mental status including confusion, irritability, and hallucinations; convulsions and seizures; cerebral edema; cerebellar herniation; then ultimately coma [8]. Death usually happens 1-18 days after the beginning of symptoms [9].

The current treatment of PAM relies on combination of deoxycholate amphotericin B and azole drug (fluconazole, voriconazole, ketoconazole), azithromycin, rifampin, and miltefosine PO [10]. A few survivors also received aggressive management of brain swelling by dexamethasone and therapeutic hypothermia [11]. Despite the use of these drugs, there have been only seven survivors worldwide to date and only four survivors from the United States [12]. Therefore, discovery of new drugs, either alone or in combination with each other, is a necessity to prevent deaths from this fatal disease.

We recently identified a few sterol biosynthesis inhibitors having superior activity to the currently used amphotericin B [13]. We also chemically validated different enzymatic chokepoints in *N. fowleri* steroidogenic pathway in vitro [14,15]. We identified FDA-approved pitavastatin as the most potent 3-hydroxy-3-methyl-glutaryl-CoA reductase (HMGR) inhibitor among all statins tested so far against *N. fowleri*. Some of the branches of mevalonate pathway produce key metabolites such as farnesyl pyrophosphate, which is important for cell metabolism [16]. Protein farnesyltransferase (FT) uses farnesyl pyrophosphate to attach farnesyl group to specific proteins and this prenylation is an important process to mediate protein-protein interactions and protein-membrane interactions [17]. Since the genome of *N. fowleri* contains a gene encoding FT (Accession number NF0130130) and compounds targeting FT of other protozoan parasites led to severe impairment of growth of *Trypanosoma cruzi*, *Leishmania major*, *Plasmodium falciparum* and *Entamoeba histolytica* [18,19,20,21], we investigated the effect of a well-characterized, late-stage clinical FT inhibitor lonafarnib against multiple strains of *N. fowleri*. We also determined the effect of the combination of pitavastatin, an HMGR inhibitor, that targets the second step of the mevalonate pathway, and the FT inhibitor lonafarnib that acts at the end of the mevalonate pathway. 

## 2. Results and Discussion

### 2.1. In Vitro Effect of Lonafarnib against Different Clinical Strains of N. fowleri 

FT, the last common enzyme for products derived from the mevalonate pathway, is vital for diverse functions including cell differentiation and growth [22]. There is evidence that targeting the FT in protozoan parasites leads to inhibition of protein prenylation and severely impairing growth [18,23,24]. FT inhibitor lonafarnib has been extensively tested in clinical trials for malignancies [25]. It has also been used in Phase I study for pediatric brain tumors [26], indicating blood–brain barrier permeability to lonafarnib. Lonafarnib has also undergone phase 3 clinical trials in children with progeria after an observational cohort study showed that lonafarnib led to a significantly reduced mortality rate after 2.2 years of follow-up [27]. Based on these clinical trial studies and on the presence of a gene encoding *N. fowleri* FT, we hypothesized that blockade of the FT by small molecule inhibitor lonafarnib would predictably be detrimental to *Naegleria* trophozoite survival.

The potency of lonafarnib varied depending on the strains of *N. fowleri* used. While both the European KUL (EC_50_ = 1.5 µM) and Australian CDC:V1005 (EC_50_ = 2.5 µM) strains were almost equally susceptible to lonafarnib (Table 1), greater variation in EC_50_ was observed among US strains. US TY strain, belonging to genotype III, was least susceptible to lonafarnib with an EC_50_ of 9.2 µM. Lonafarnib showed about 2- to 2.5-fold better potency against US Davis (EC_50_ = 5 µM) and CAMP (EC_50_ = 3.5 µM) strains, respectively than the TY strain (Table 1).

### 2.2. Effect of Combination of Lonafarnib and Pitavastatin on N. fowleri

Since HMGR and FT are both involved in the cholesterol biosynthesis pathway, we investigated if the effect of the combination of the most potent anti-*Naegleria* HMGR inhibitor and lonafarnib would cause more deleterious effect on the trophozoites than the single drug. Recently, we identified blood–brain barrier permeable pitavastatin as a potent amebicidal against the US, Australian and European strains of *N. fowleri*. Pitavastatin was equipotent to the standard of care amphotericin B and exhibited an EC_50_ of 0.3 µM against the European KUL strain. Pitavastatin, an FDA-approved HMGR inhibitor, targets the HMG-CoA reductase of the mevalonate pathway. Since both lonafarnib and pitavastatin target two essential enzymes of the mevalonate pathway and successful treatment of *Naegleria* infection often requires combination therapy [11], we explored the effect of the combination of lonafarnib and pitavastatin on *N. fowleri* trophozoites.

The inhibitory effects of lonafarnib and pitavastatin were determined at fixed concentration ratios and their dose-effect relationships were assessed by Chou-Talalay combination index (CI) method [28] using CompuSyn software. The growth inhibition was estimated by ATP-bioluminescence assay and results provided a quantitative determination for synergism (CI < 1), additivity (CI = 1), and antagonism (CI > 1). Combination of lonafarnib and pitavastatin in a ratio of 1:1, 1:2 1:4, 1:8, 1:16, 2:1, 4:1, 8:1 and 16:1 elicited synergistic activity with CI values ranging between 0.29 and 0.73 and caused 95% growth inhibition of *N. fowleri* trophozoites (Table 2). When combined, a dose reduction of about 5- to 30-fold for pitavastatin and about 2- to 28-fold for lonafarnib was achieved at different drug ratios to inhibit 95% growth of the trophozoites (Table 2).

Both lonafarnib and pitavastatin are well-tolerated and the maximum recommended dose of FDA-approved pitavastatin is 4 mg. Several Phase I studies in cancer patients identified the maximum tolerated dose of lonafarnib as 200 mg b.i.d. [29]. Lonafarnib was also found well-tolerated in different Phase I clinical combination studies [29]. The side effects associated with the use of lonafarnib were mainly gastrointestinal [29]. The advantage of using lonafarnib and pitavastatin in PAM is that both have pediatric formulations available. Moreover, pitavastatin exhibited blood–brain barrier permeability in the cell culture [30] and lonafarnib was tested in a Phase I clinical trial in recurrent or progressive pediatric brain tumors [26]. These properties make the combination of lonafarnib and pitavastatin an attractive strategy for the development of future therapy for PAM.

### 2.3. Microscopic Evaluation to Determine the Effect of Combination of Lonafarnib and Pitavastatin at Different Time Points

**Figure 1:**
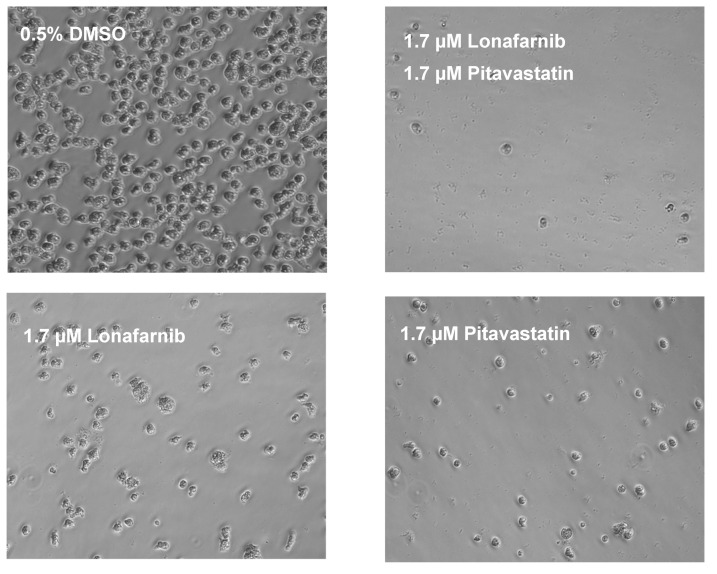
Synergistic effect of lonafarnib and pitavastatin at 24 h. The phase contrast microscope images show *N. fowleri* KUL trophozoites treated for 24 h with 0.5% DMSO, 1.7 µM of lonafarnib, 1.7 µM of pitavastatin, and a combination of 1.7 μM of lonafarnib and 1.7 µM of pitavastatin. Trophozoites treated with lonafarnib-pitavastatin pair are rounded, and much smaller in size, whereas DMSO-treated cells are irregularly shaped with visible cytoplasm. Magnification, ×20.

**Figure 2:**
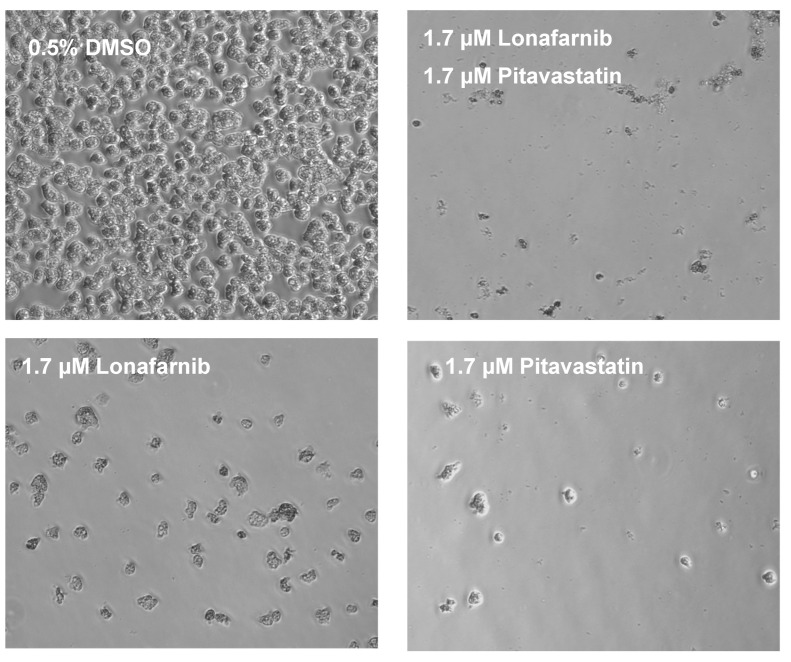
Synergistic effect of lonafarnib and pitavastatin at 48 h. The phase contrast microscope images show *N. fowleri* KUL trophozoites treated for 48 h with 0.5% DMSO, 1.7 µM of lonafarnib, 1.7 µM of pitavastatin, and a combination of 1.7 μM of lonafarnib and 1.7 µM of pitavastatin. No trophozoite with normal morphology is observed when treated with lonafarnib-pitavastatin pair, whereas DMSO-treated cells are irregularly shaped with visible cytoplasm. Magnification, ×20.

Synergistic activities of lonafarnib and pitavastatin were achieved at different drug ratios. To confirm the computationally calculated synergism, we independently performed a drug combination study and analyzed its effect microscopically. Although CompuSyn software identified that a combination of 3.1 µM of lonafarnib and 3.1 µM of pitavastatin (1:1) caused 95% growth inhibition of *N. fowleri* trophozoites (Table 2), we tested a lower concentration of both drugs maintaining a 1:1 ratio. Thus, we selected a combination of 1.7 µM of lonafarnib and 1.7 µM of pitavastatin and compared its effect on *N. fowleri* trophozoites to respective concentration of the drug alone. The effect was determined at 24 and 48 h. While 1.7 µM of lonafarnib alone and same concentration of pitavastatin alone decreased the growth of trophozoites at 24 h, the effect was more pronounced when both drugs were combined at 1:1 ratio. Very few trophozoites were observed under microscope at this time point with lonafarnib-pitavastatin pair of 1.7 µM each and the remaining trophozoites were much smaller in size and rounded in shape (Figure 1). The effect on trophozoites at 48 h was more prominent than the effect at 24 h. Although a combination of 1.7 µM of lonafarnib and 1.7 µM of pitavastatin resulted in fewer trophozoites at 24 h, no trophozoite with normal morphology was observed at 48 h in presence of a combination of 1.7 µM of lonafarnib and 1.7 µM of pitavastatin (Figure 2).

## 3. Materials and Methods

### 3.1. Maintenance of N. fowleri Culture 

The trophozoites of the reference *N. fowleri* European KUL strain (ATCC 30808), the US clinical strains Davis, CAMP, and TY (acquired from CDC) and the Australian CDC:V1005 strain (acquired from CDC) were maintained axenically in Nelson medium supplemented with 10% fetal bovine serum at 37 °C [13]. US clinical strains Davis, CAMP, and TY belonged to genotypes I, II and III, respectively. The high virulence of the KUL strain was maintained by infecting BALB/c mice periodically with KUL trophozoites and by harvesting brain tissues to recover parasites. These recovered highly pathogenic KUL trophozoites were used in the experiments. All experiments used trophozoites harvested at 48 h during the logarithmic phase of growth. 

### 3.2. In Vitro Effect of Lonafarnib against Different Clinical Strains of N. fowleri

Lonafarnib (Sigma) was tested for its activity against European KUL; US Davis, CAMP, TY; and Australian CDC:V1005 strains of *N. fowleri*. Lonafarnib was dissolved as 20 mM stock in DMSO. Starting at 50 μM, the compound was serially diluted with DMSO to eight concentrations. 0.5 μL from each concentration was transferred in triplicate into a flat white bottom 96 well microtiter plate. 0.5% DMSO was used as a vehicle control and 50 μM of amphotericin B as a positive control. Density of 10,000 *N. fowleri* trophozoites per 99.5 μL was seeded across the plate. In between each transfer, the cells were evenly resuspended throughout the medium. After 48 h of incubation at 37 °C, the plates were taken out and cooled to room temperature. CellTiter-Glo^®^ Luminescent Cell Viability Assay (Promega Corporation, Madison, WI, USA) was utilized to measure generation of ATP-bioluminescence [31]. The assay solution contains luciferase, which in the presence of cellular ATP and oxygen, catalyzes the transformation of luciferin into oxyluciferin, yielding PPi, AMP, and light. The luminescence was quantified using EnVision Multilabel plate reader (PerkinElmer, Inc. Waltham, MA, USA). Microsoft Excel and GraphPad Prism 5.0 (GraphPad Software, San Diego, CA, USA) softwares were used for statistical analysis of experiments and determination of EC_50_ of lonafarnib.

### 3.3. Effect of Combination of Lonafarnib and Pitavastatin on N. fowleri

Recently, we found that pitavastatin was the most potent statin against *N. fowleri* and, therefore, the effect of combining pitavastatin with lonafarnib against *N. fowleri* trophozoites was investigated. 5 mM pitavastatin (Selleck Chemicals) was prepared from a 20-mM stock concentration, and 0.25 μL was transferred into combination wells. Then, 0.25 μL of lonafarnib from 20-mM stock was added to the same well with pitavastatin and maintained a final 0.5% DMSO. Starting concentrations were established at the lowest concentrations that induced 100% inhibition and serially diluted to eight concentrations. The starting concentrations of lonafarnib and pitavastatin were 50 μM and 12.5 μM, respectively. Concentration of lonafarnib changed with column, while that of pitavastatin changed with row, in a checkerboard fashion. Each plate contained a column of different concentrations of lonafarnib and another column of different concentrations of pitavastatin to determine the EC_50_ of individual drugs. Negative control included 0.5% DMSO and positive control included 50 µM of amphotericin B. The experiments were performed in triplicate and after adding 10,000 KUL trophozoites into 99.5 μL of culture medium in each well, the plates were incubated for 48 h 37 °C. The cell viability was measured by CellTiter-Glo and the effect of combination was determined by Chou-Talalay Combination Index method using CompuSyn software [28].

### 3.4. Microscopic Evaluation to Determine the Effect of Combination of Lonafarnib and Pitavastatin at Different Time Points

To confirm the synergistic activity calculated computationally by CompuSyn and to determine the effect of combination of lonafarnib and pitavastatin on the phenotype of *N. fowleri*, we performed an experiment in a 96-well clear bottom plate with 10,000 *N. fowleri* European KUL trophozoites in each well. Trophozoites were incubated with 1.7 µM of lonafarnib and 1.7 µM of pitavastatin, both alone and in combination for 24 and 48 h at 37 °C. Trophozoites treated with 0.5% DMSO were used as a negative control. All experiments were performed in triplicate. Trophozoites were imaged at 24 and 48 h under Zeiss Axiovert 40 CFL phase contrast microscope to understand the effect of pitavastatin and lonafarnib, alone and in combination, on the phenotype of trophozoites.

## 4. Conclusions

Taken together, we report the potent anti-*Naegleria* activity of the clinically developed FT inhibitor lonafarnib. The potency increased when lonafarnib was combined with the FDA-approved HMGR inhibitor pitavastatin. Blood–brain barrier may exhibit permeability to both pitavastatin and lonafarnib and pediatric formulations are available for both the drugs. Future studies will involve the testing of lonafarnib against the FT of *N. fowleri*. The synergistic activity of the combination of lonafarnib and pitavastatin against *N. fowleri* provides an opportunity to investigate a combination therapy in the animal model of PAM.

## Figures and Tables

**Table 1:** EC_50_ values of lonafarnib against trophozoites of different strains of *N. fowleri.*

Compound	Strain	EC_50_ (µM) Mean ± SE
Lonafarnib	KUL	1.5 ± 0.1
	CDC:V1005	2.5 ± 0.04
	US Davis (Genotype I)	5 ± 0.09
	US CAMP (Genotype II)	3.5 ± 0.05
	US TY (Genotype III)	9.2 ± 0.05

**Table 2:** Summary of synergism assay with lonafarnib and pitavastatin, shown for 95% growth inhibition of *N. fowleri* trophozoites.

Lonafarnib:Pitavastatin Ratio	% Growth Inhibition of *N. fowleri* KUL Trophozoites	Combination Index (CI)	Dose Reduction Index (DRI)		Dose Required to Achieve 95% Inhibition (µM)	
			Lonafarnib	Pitavastatin	Lonafarnib	Pitavastatin
1:1	95	0.36	5.1	6.1	3.1	3.1
1:2	95	0.29	9.2	5.5	1.7	3.4
1:4	95	0.29	14.9	4.5	1	4.2
1:8	95	0.38	19.9	3	0.8	6.2
1:16	95	0.46	31.4	2.4	0.5	7.9
2:1	95	0.38	3.8	9	4.1	2.1
4:1	95	0.53	2.3	11.1	6.8	1.7
8:1	95	0.59	1.9	18.1	8.3	1
16:1	95	0.73	1.4	27.6	10.8	0.7
